# Nickel Nanoparticles cause exaggerated lung and airway remodeling in mice lacking the T-box transcription factor, TBX21 (T-bet)

**DOI:** 10.1186/1743-8977-11-7

**Published:** 2014-02-06

**Authors:** Ellen E Glista-Baker, Alexia J Taylor, Brian C Sayers, Elizabeth A Thompson, James C Bonner

**Affiliations:** 1 Environmental & Molecular Toxicology Program, Department of Biological Sciences, North Carolina State University, Campus Box 7633, Raleigh, North Carolina 27695, USA

**Keywords:** Nickel nanoparticles, Carbon nanotubes, T-bet, Asthma, Lung injury

## Abstract

**Background:**

Nickel nanoparticles (NiNPs) are increasingly used in a variety of industrial applications, including the manufacturing of multi-walled carbon nanotubes (MWCNTs). While occupational nickel exposure is a known cause of pulmonary alveolitis, fibrosis, and cancer, the health risks of NiNPs are not well understood, especially in susceptible individuals such as asthmatics. The T-box transcription factor Tbx21 (T-bet) maintains Th1 cell development and loss of T-bet is associated with a shift towards Th2 type allergic airway inflammation that characterizes asthma. The purpose of this study was to determine the role of T-bet in susceptibility to lung remodeling by NiNPs or MWCNTs.

**Methods:**

Wild-type (WT) and T-bet^-/-^ mice were exposed to NiNPs or MWCNTs (4 mg/kg) by oropharyngeal aspiration (OPA). Necropsy was performed at 1 and 21 days. Bronchoalveolar lavage fluid (BALF) was collected for differential counting of inflammatory cells and for measurement of cytokines by ELISA. The left lung was collected for histopathology. The right lung was analyzed for cytokine or mucin (MUC5AC and MUC5B) mRNAs.

**Results:**

Morphometry of alcian-blue/periodic acid Schiff (AB/PAS)-stained lung tissue showed that NiNPs significantly increased mucous cell metaplasia in T-bet^-/-^ mice at 21 days (p < 0.001) compared to WT mice, and increased MUC5AC and MUC5B mRNAs (p < 0.05). MWCNTs also increased mucous cell metaplasia in T-bet^-/-^ mice, but to a lesser extent than NiNPs. Chronic alveolitis was also increased by NiNPs, but not MWCNTs, in T-bet^-/-^ mice compared to WT mice at 21 days (P < 0.001). NiNPs also increased IL-13 and eosinophils (p < 0.001) in BALF from T-bet^-/-^ mice after 1 day. Interestingly, the chemokine CCL2 in the BALF of T-bet^-/-^ mice was increased at 1 and 21 days (p < 0.001 and p < 0.05, respectively) by NiNPs, and to a lesser extent by MWCNTs at 1 day. Treatment of T-bet^-/-^ mice with a monoclonal anti-CCL2 antibody enhanced NiNP-induced mucous cell metaplasia and MUC5AC mRNA levels (p < 0.05), yet marginally reduced NiNP-induced alveolitis.

**Conclusion:**

These findings identify T-bet as a potentially important susceptibility factor for NiNP exposure and to a lesser extent for MWCNT exposure, and suggests that individuals with asthma are at greater risk.

## Background

Asthma is a chronic inflammatory disease of the airways that currently affects more than 25 million people in the United States and 300 million people worldwide [1]. It is characterized by periods of acute bronchoconstriction defined by airway hyperresponsiveness, mucus hypersecretion, and airway remodeling that involves eosinophilic inflammation, subepithelial collagen deposition, airway smooth muscle cell hypertrophy and hyperplasia, and mucous cell metaplasia (a phenotypic shift of the mucus-producing airway epithelium from serous cells to mucus-laden goblet cells) [2]. Exposure to allergens, viral infections, and environmental and occupational irritants, such as particulate matter, often trigger asthma exacerbations that lead to airflow obstruction through exaggerated inflammation and increased mucus production [3]. Ultrafine particulate matter <100 nm (i.e., nanoparticles) are of concern in the exacerbation of asthma as they can reach more distal areas of the lung and possess greater surface area per unit mass as compared to larger particles [4].

Environmental, occupational, and genetic factors are thought to act together to initiate allergen-mediated airway inflammation through T cell activation [5]. Early studies found increased numbers of T helper 2 (Th2) cells in the airways of asthmatics and defined them as the key players in regulating allergic lung inflammation. In turn, Th2 cells secrete elevated levels of the cytokines IL-4, IL-5, and IL-13 through the transcription factor GATA-3 that initiates and maintains the inflammatory response [6,7]. The T-box transcription factor TBX21 (T-bet), maintains Th1 cell differentiation in the lung and regulates the production of IFN-γ while inhibiting the development of Th2 cells [8-10]. Mice with a targeted deletion of T-bet (T-bet^-/-^) have decreased amounts of IFN-γ and overproduce Th2 cytokines [8]. The lungs of T-bet^-/-^ mice have been reported to display spontaneous airway remodeling, subepithelial collagen deposition, IL-13 mediated airway hyper-reactivity (AHR) and eosinophil infiltration, similar to an asthma-like phenotype [9]. Interestingly, the airways of asthmatics have been shown to have significantly less T-bet expression when compared to non-asthmatic lungs suggesting that T-bet^-/-^ mice would be a useful Th2-mediated model of asthma [10].

The rapidly growing nanotechnology industry has led to the manufacture of an increasing variety of novel engineered nanomaterials (ENMs). Because of their unique chemical and physical properties, ENMs could interact with biological systems in ways that differ from that of bulk materials composed of the same elemental composition, thus preventing the ability to accurately predict how ENMs will impact human health and the environment [4,11,12]. The inhalation of nickel (Ni), particularly in occupational settings, acutely causes inflammation and allergic reactions, while chronic exposure to Ni has been linked to pulmonary fibrosis, chronic alveolitis and lung cancer [13,14]. Therefore, it is likely that nickel nanoparticles (NiNPs) could pose similar threats to human health, yet information on human disease caused by NiNPs is lacking. We and others have shown that NiNPs are potent activators of mitogen-activated protein kinases (MAPK) and hypoxia-inducible factor (HIF)-1α in pleural mesothelial cells or lung epithelial cells [15,16]. NiNP and Ni-based nanoparticles are also more toxic than micron-sized Ni particles [16]. Moreover, NiNP compounds cause greater pulmonary inflammation and toxicity in the lungs of rodents when directly compared to Ni particle counterparts [17-19].

NiNPs are widely used as catalysts for the manufacture of multi-walled carbon nanotubes (MWCNTs). We have shown that MWCNTs manufactured with a NiNP catalyst exacerbate airway fibrosis in mice with pre-existing allergic lung inflammation, suggesting that these ENMs pose a potential health risk for susceptible individuals with asthma [20]. More recently, we demonstrated that susceptibility to MWCNT-induced airway inflammation following allergen challenge was enhanced by the loss of cyclooxygenase-2 (COX-2), indicating that the loss of specific genes could be important determinants in susceptibility to ENMs [21]. In this study we analyzed the effects of NiNPs on the exacerbation of allergic lung inflammation in T-bet^-/-^ mice, a genetically modified mouse model of Th2-mediated allergic lung inflammation. We found that NiNPs increased mucous cell metaplasia in T-bet^-/-^ mice 21 days after initial exposure but not in wild-type (WT) mice. Additionally, mediators of mucus regulation, IL-13 and CCL2, as well as eosinophil infiltration, were increased in T-bet^-/-^ mice in response to NiNP. Interestingly, chronic alveolitis a hallmark response of occupational nickel exposure, was enhanced in T-bet^-/-^ mice. Our findings suggest that individuals with T-bet deficiency are more susceptible to allergic airway remodeling or chronic alveolitis from NiNP exposure.

## Results

### Mucous cell metaplasia is amplified in T-bet deficient mice after NiNP exposure

**Figure 1:**
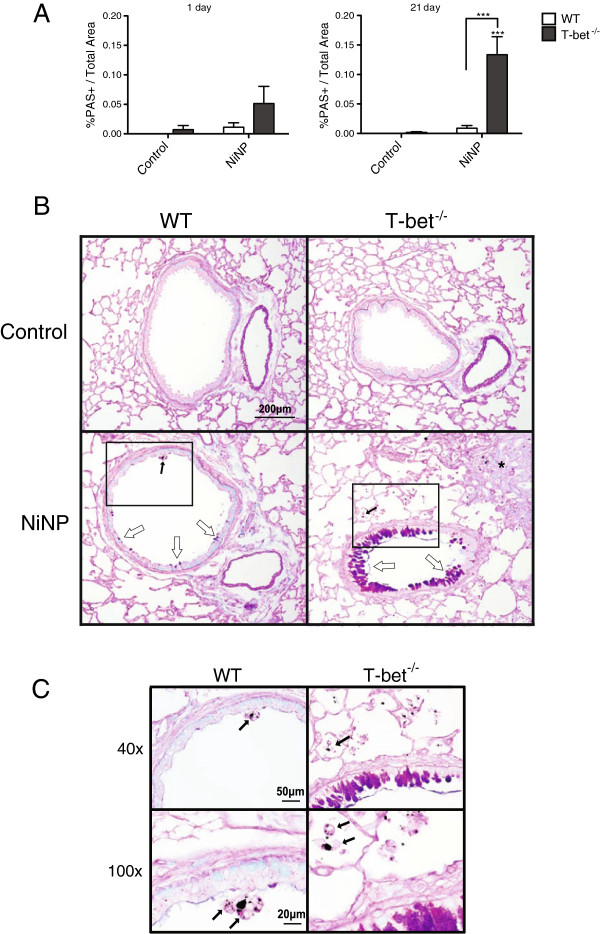
**Mucous cell metaplasia in response to NiNP exposure in WT and T-bet**^**-/- **^**mice. A)** Quantification of mucus producing cells at 1 or 21 days post-exposure determined using ImageJ analysis software (NIH). Data presented as the percentage of AB/PAS-positive stained area per total area. ***p < 0.001 compared to the control group of the same genotype or as indicated. All data represent mean values ± SEM of at least three measurements per lung of 5–7 mice per exposure group. **B)** Low magnification (10×) of AB/PAS stained lung tissue sections (open arrows) at 21 days after WT and T-bet^-/-^ mice were exposed to a 0.1% pluronic solution or NiNP. Asterisk indicates area of fibrosis. **C)** High magnification (40× and 100×) of insert from 10× in **(*B*)**. Black arrows indicate alveolar macrophages containing NiNP agglomerates.

Wild-type (WT) and T-bet knockout (T-bet^-/-^) mice were exposed to nickel nanoparticles (NiNPs) or multi-walled carbon nanotubes (MWCNTs) by oropharyngeal aspiration (OPA) and lung tissue was harvested 1 day or 21 days after initial exposure. The effect of NiNPs on mucous cell metaplasia was evaluated first using semi-quantitative scoring of AB/PAS-stained lung sections as described in Materials & Methods (%PAS+/Total Area) to identify the relative area of airway epithelium represented by mucin-producing goblet cells. Data from these measurements showed that NiNP exposure increased mucous cell metaplasia in WT or T-bet^-/-^ mice at 1 day, although this increase was not significant compared to controls or between genotypes (Figure 1A). After 21 days, NiNPs caused a robust and highly significant increase in mucous cell metaplasia in T-bet^-/-^ mice, while WT mice exposed to NiNPs showed only a slight increase in mucous cell metaplasia that was not statistically significant (Figure 1A). Photomicrographs of AB/PAS-stained lung sections portraying representative airways at 21 days post exposure showed relatively few goblet cells in the airways of WT mice after exposure to NiNPs, indicating relatively weak mucous cell metaplasia (Figure 1B). However, in T-bet^-/-^ mice, mucous cell metaplasia dramatically increased after NiNP exposure (Figure 1B). Higher magnification of the airways revealed alveolar macrophages containing NiNPs in close proximity to goblet cells in both genotypes (Figure 1C). MWCNTs also significantly increased mucous cell metaplasia in the lungs of T-bet^-/-^ mice, but to a significantly lesser extent as that observed with NiNPs (Additional file 1A).

### MUC5AC and MUC5B mRNA levels are increased by NiNP in T-bet^-/-^ mice

**Figure 2:**
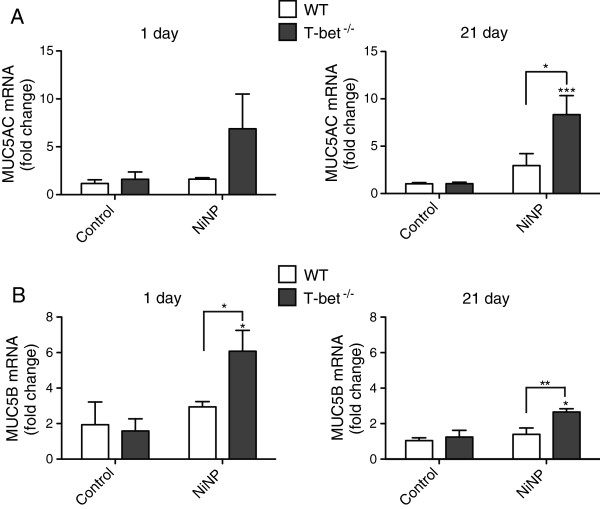
**MUC5AC and MUC5B mRNA levels at 1 and 21 days after exposure.** Taqman quantitative real-time RT-PCR was used to measure changes in whole lung mRNA levels of **A)** MUC5AC and **B)** MUC5B. Values are means ± SEM (*n* = 5–7 animals/group). *p < 0.05, **p < 0.01, ***p < 0.001 as compared to the control group of the same genotype or as indicated.

Since mucous cell metaplasia was increased in T-bet^-/-^ mice in response to NiNPs, we measured MUC5AC and MUC5B mRNA expression levels in whole lung tissue. Levels of MUC5AC mRNA were increased in the lungs of T-bet^-/-^ mice one day after NiNP exposure, albeit not to a significant extent. However, MUC5AC mRNA levels were significantly increased in T-bet^-/-^ mice at 21 days after NiNP exposure compared to NiNP-treated WT mice (Figure 2A). MUC5B mRNA in whole lung tissue was also significantly increased at 1 day and 21 days post NiNP exposure in T-bet^-/-^ mice compared to NiNP-treated WT mice (Figure 2B). NiNPs did not have a significant effect on MUC5AC or MUC5B mRNA expression in WT mice at either time point. The Taqman quantitative RT-PCR results shown in Figure 2 supported the semi-quantitative pathology scoring of mucous cell metaplasia in T-bet^-/-^ mice exposed to NiNPs (Figure 1). MWCNTs also increased MUC5AC mRNA levels 2 to 3-fold in the lungs of T-bet^-/-^ mice, although this increase was not statistically significant compared to saline control and was significantly lower than the induction of MUC5AC mRNA by NiNPs (Additional file 1B).

### T-bet^-/-^ mice display spontaneous eosinophilia that is enhanced by NiNPs

**Figure 3:**
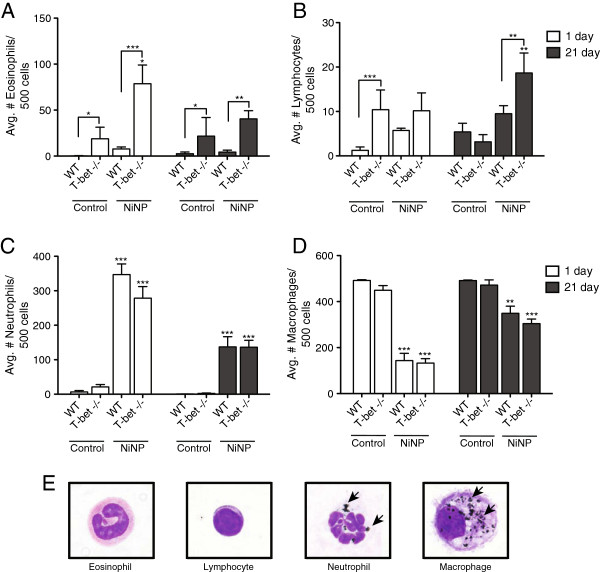
**Differential cell counts in the BALF 1 and 21 day after NiNP exposure.** Immune cells numbers for **A)** eosinophils, **B)** lymphocytes, **C)** neutrophils, and **D)** macrophages are presented as the mean values ± SEM out of a total of 500 cells counted per animal for 5–7 animals per dose group at 20× magnification. *p < 0.05, **p < 0.01, ***p < 0.001 compared to the time matched control group of the same genotype or as indicated. **E)** Photomicrographs representing each cell type. Macrophages and neutrophils demonstrated phagocytosis of NiNP in both genotypes at both time points in response to exposure (100×).

Numbers of eosinophils in BALF were spontaneously increased at 1 and 21 days in the BALF of all T-bet^-/-^ mice compared to WT mice (Figure 3A). Furthermore, NiNP exposure caused an exaggerated and transient increase in eosinophils in T-bet^-/-^ mice 1 day after treatment. In comparison to NiNPs, MWCNTs did not significantly increase numbers of eosinophils above 0.1% pluronic control in T-bet^-/-^ mice at 1 day (Additional file 2A). Lymphocyte counts were also slightly higher in the BALF of T-bet^-/-^ mice at 1 day and significantly increased in response to NiNP exposure by 21 days (Figure 3B). However, the overall number of lymphocytes remained low in both WT and T-bet^-/-^ mice in comparison to other inflammatory cell types. NiNP exposure increased numbers of neutrophils in BALF after 1 day in either WT or T-bet^-/-^ mice (Figure 3C). MWCNTs also significantly increased numbers of neutrophils in BALF in both WT and T-bet^-/-^, although to a lesser extent compared to NiNP exposure (Additional file 2B). Neutrophil number decreased by 21 days but still remained significantly elevated in NiNP-treated WT and T-bet^-/-^ mice (Figure 3C). Conversely, NiNP exposure decreased the relative number of macrophages similarly in WT and T-bet^-/-^ mice (Figure 3D). Representative photomicrographs of each inflammatory cell type taken from Diff-Quik®-stained cytospins of BALF collected from WT and T-bet^-/-^ mice at 21 days showed the cytoplasm of macrophages and neutrophils contained numerous NiNPs, whereas no NiNPs were found in lymphocytes or eosinophils (Figure 3E). A similar level of NiNPs was observed in the lungs of WT mice compared to T-bet^-/-^ mice, although we did not quantify absolute levels of NiNPs in lung tissue or BALF cells.

### Inflammation is elevated in the lungs of WT and T-bet^-/-^ mice exposed to NiNPs

**Figure 4:**
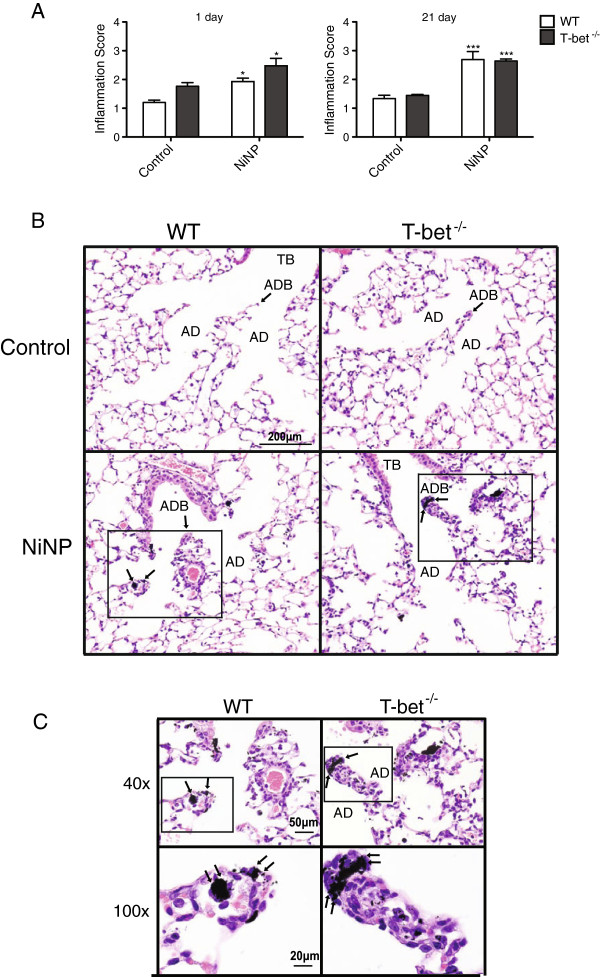
**Histopathological analysis of inflammation in the lungs of WT and T-bet**^**-/- **^**mice in response to NiNPs. A)** Lung pathology was scored in mice for inflammation 1 and 21 days after initial exposure. All data represent mean values ± SEM. *p < 0.05, ***p < 0.001 compared to the control group of the same genotype (*n* = 5–7 animals/group). **B)** Representative photomicrographs at low magnification (10×) of H&E stained lung sections at 21 days after mice were exposed. **TB**: Terminal bronchiole, **ADB**: alveolar duct bifurcation, **AD**: alveolar duct. **C)** Higher magnification (40× and 100×) of images from 10× in **(B)**.

Inflammation of the lungs of WT and T-bet^-/-^ mice was evaluated by semi-quantitative scoring of H&E-stained lung sections. Inflammatory scores revealed a significant increase in both genotypes following NiNP exposure at 1 and 21 days post exposure when compared to their respective controls (Figure 4A). At 1 day, histopathology showed infiltration of inflammatory cells and thickening of alveolar walls and alveolar duct bifurcations (ADB) in response to NiNPs in WT and T-bet^-/-^ mice (Figure 4B). Additionally, these structural changes were also seen at the 21 day time point in NiNP treated mice (data not shown). Upon further evaluation, higher magnification revealed deposition of NiNPs at ADB as well as NiNPs contained within alveolar macrophages (Figure 4C).

### Airway fibrosis is increased similarly in WT and T-bet^-/-^ mice exposed to NiNPs, yet NiNP-induced alveolitis is exaggerated in T-bet^-/-^ mice

**Figure 5:**
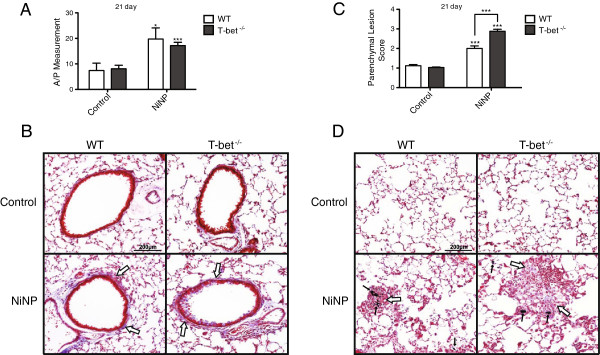
**The effects of NiNP on airway fibrosis and parenchymal alveolitis in WT and T-bet**^**-/- **^**mice. A)** Cross-sections of airways stained with trichrome were measured for the area to perimeter ratio of collagen deposition. **B)** Representative images of results from **(A)** of airways stained with trichrome (open arrows) at low magnification (10×) at 21 days post exposure. **C)** Lung pathology was then scored for parenchymal lesions in WT and T-bet^-/-^ mice. **D)** Low magnification (10×) of trichrome stained lung parenchyma sections (open arrows) at 21 days after WT and T-bet^-/-^ mice were initially exposed. Images are a representation of the data shown in **(C)**. Black arrows indicate NiNP aggregates. Data are the mean values ± SEM (*n* = 5–7 animals/group). *p < 0.05, ***p < 0.001 compared to the control group of the same genotype or as indicated.

Airway fibrosis, another characteristic associated with the pathogenesis of allergic asthma, was measured at 21 days after NiNP exposure by performing a semi-quantitated morphometric analysis using Adobe Photoshop (Figure 5A). NiNP exposure significantly increased the thickness of airway walls as determined by quantitative morphometry in both WT and T-bet^-/-^ mice when compared to their respective controls (Figure 5A and B). Both WT and T-bet^-/-^ mice displayed parenchymal lesions containing inflammatory cells and extracellular matrix components within alveolar spaces along with alveolar wall thickening (i.e., alveolitis) and typically associated with NiNPs. However, the number and size of interstitial lesions, collectively termed ‘parenchymal lesion score’, were significantly increased in T-bet^-/-^ mice compared to WT after NiNP exposure at 21 days (Figure 5C). The representative images from trichrome-stained lung sections shown in Figure 5D depict the differences in parenchymal lesions seen between WT mice and T-bet^-/-^ mice. Mice that received an OPA dose of 0.1% pluronic solution did not display any parenchymal lesions and were scored as a “1”. NiNP or MWCNT exposure did not cause a significant increase in whole lung col1a2 mRNA expression measured by Taqman® quantitative RT-PCR. Moreover, NiNPs caused no increase in lung soluble collagen protein measured by Sircol® Assay in T-bet^-/-^ or WT mice (Additional file 3).

### NiNP-induced CCL2 mRNA and protein expression is enhanced in T-bet^-/-^ mice

**Figure 6:**
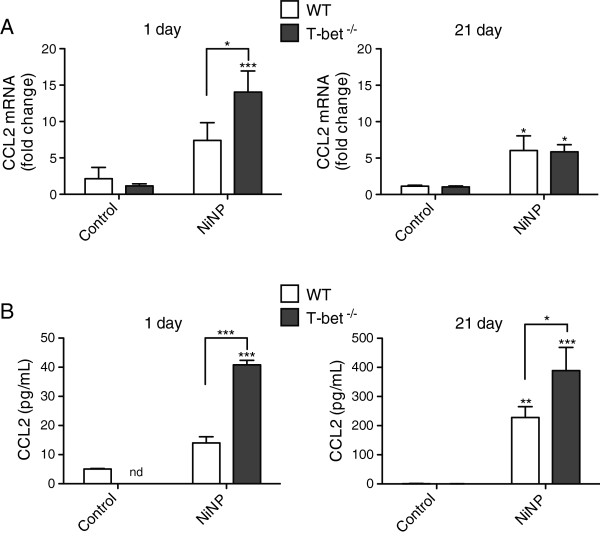
**CCL2 mRNA and protein levels in the lungs of mice after NiNP exposure. A)** CCL2 mRNA expression was measured by qRT-PCR in whole lung tissue at 1 and 21 days post exposure while **B)** CCL2 protein in BALF was analyzed by ELISA after 1 or 21 days of initial exposure (nd, not detectable). *p < 0.05, **p < 0.01, ***p < 0.001 as compared to the control group of the same genotype or as indicated. Data are the mean values ± SEM (*n* = 5–7 animals/group).

We measured CCL2 expression since it has been implicated in mucin expression as well as lung inflammation and fibrogenesis. CCL2 mRNA expression in whole lung tissue measured by qRT-PCR was slightly increased by NiNP exposure after 1 day in WT mice. However, a significant increase in NiNP-induced CCL2 mRNA levels was observed in T-bet^-/-^ mice compared to WT mice (Figure 6A). By 21 days, overall CCL2 mRNA levels had decreased but were still significant in both genotypes as compared to their time-matched controls (Figure 6A). CCL2 protein levels measured in BALF mirrored that of CCL2 mRNA expression at 1 day post-exposure as T-bet^-/-^ mice had significantly more CCL2 protein than WT mice exposed to NiNPs (Figure 6B). By 21 days, the amount of CCL2 protein dramatically increased (~10-fold) in both WT and T-bet^-/-^ mice in response to NiNPs compared to the amount of protein that was expressed in the BALF at 1 day (Figure 6B). Furthermore, CCL2 protein levels at 21 days were significantly enhanced in T-bet^-/-^ mice compared to WT mice, suggesting that the presence of T-bet not only inhibits mucous cell metaplasia but CCL2 mRNA and protein as well. MWCNTs also significantly increased protein levels of CCL2 in the BALF from T-bet^-/-^ mice, but to a significantly lesser extent when compared to NiNP exposure (Additional file 4). Additionally, levels of IL-13, a well-known regulator of mucous cell metaplasia and inflammation in Th2-mediated asthma, were measured in whole lung tissue and BALF for mRNA and protein expression, respectively. IL-13 mRNA was increased in response to NiNPs at 1 day post exposure in both WT and T-bet^-/-^ mice, yet only IL-13 protein was increased in T-bet^-/-^ mice after NiNP exposure at 1 day (Additional file 5). MWCNTs did not cause an increase in IL-13 levels in BALF at 1 or 21 days post-exposure (data not shown).

### Anti-CCL2 enhances NiNP-induced mucous cell metaplasia in T-bet^-/-^ mice

**Figure 7:**
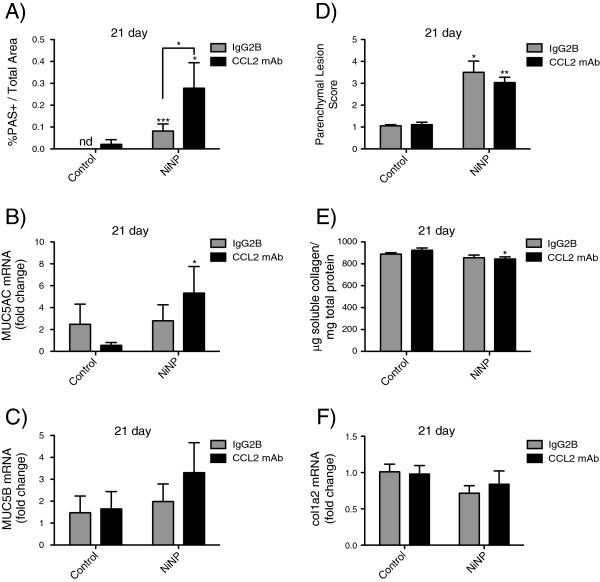
**Mucous cell metaplasia and alveolitis in response to anti-CCL2 mAb treatment in T-bet**^**-/- **^**mice 21 days post-exposure. A)** Cells stained with AB/PAS were quantitated for mucin protein expression using ImageJ (NIH) analysis for percentage of positive stained area per total area in mice treated with IgG2B Isotype Control or anti-CCL2 mAb and exposed to either a 0.1% pluronic solution or NiNPs. **B)** MUC5AC and **C)** MUC5B whole lung mRNA expression were quantitated using qRT-PCR analysis. **D)** Cross sections of lungs stained with trichrome were scored for parenchymal lesions in WT and T-bet^-/-^ mice 21 days after initial NiNP exposure. Lungs were scored in a blinded manner by three independent reviewers. **E)** Soluble collagen content was measured using the Sircol Assay kit in whole lung homogenates and expressed as μg/mg of protein. **F)** Whole lung col1a2 mRNA expression measured by qRT-PCR. *p < 0.05, **p < 0.01, ***p < 0.001 compared to the time matched control group of the same genotype or as indicated. Data are the mean values ± SEM (*n* = 3–5 animals/group).

The role of CCL2 in mediating NiNP-induced mucous cell metaplasia and alveolitis was analyzed by blocking CCL2 activity using a monoclonal antibody. This experiment was performed with T-bet^-/-^ mice that were treated with sequential i.p. injections of either an IgG2B isotype control mAb or anti-CCL2 monoclonal neutralizing (mAb) prior to and after exposure to a single dose of NiNPs by oropharyngeal aspiration. Necropsy was performed only at 21 days after NiNP exposure. Lung sections were stained with AB/PAS to Quantify* mucous cell metaplasia using the ImageJ method as previously described above. Surprisingly, T-bet^-/-^ mice treated with an anti-CCL2 mAb had exaggerated NiNP-induced mucous cell metaplasia (Figure 7A). Anti-CCL2 mAb also increased NiNP-induced MUC5AC and MUC5B mRNA levels in T-bet^-/-^ mice (Figure 7B and C). Additionally, at 21 days alveolitis was analyzed since there was a significant difference between genotypes exposed to NiNPs (Figure 5C and D). Although not significant, T-bet^-/-^ mice exposed to NiNPs and treated with anti-CCL2 mAb reduced the parenchymal lesion score compared to T-bet^-/-^ mice treated with IgG2B Isotype Control mAb (Figure 7D). Conversely, soluble collagen measured by the Sircol Assay was not different between isotype and anti-CCL2 mAb treatments (Figure 7E). A small group of WT mice were exposed to either a 0.1% pluronic solution (n = 2) or NiNPs (n = 3) without any mAb treatments in order to act as a repeat of the initial *in vivo* experiment. All data analyzed with this group reproduced the previous results showing that NiNP-induced mucous cell metaplasia and alveolitis were significantly less in WT mice compared to T-bet^-/-^ mice (data not shown).

## Discussion

Engineered nanoparticles, including metal catalysts such as Ni, are increasingly used for industrial purposes. Similar to micron-sized nickel particles, the major route of exposure to nickel nanoparticles (NiNPs) is through inhalation exposure [13,22,23]. Individuals with pre-existing allergic lung disease or individuals with deficiencies in specific genes, which serve to suppress allergic sensitization, would presumably be at greater risk. In this study, we investigated the effect of NiNPs or MWCNTs in mice lacking T-bet, a transcription factor that serves to suppress allergic inflammation by maintaining a Th1 immune response and preventing the development of a Th2 immune response that is typical of diseases such as asthma [8-10]. T cells in T-bet^-/-^ mice are polarized towards a Th2 phenotype, which mediate allergic airway remodeling. We found that T-bet^-/-^ mice exposed to NiNPs had exaggerated airway mucous cell metaplasia, higher levels of mucin gene mRNAs, and increased levels of IL-13 and CCL2, two cytokines implicated in allergic asthma. Moreover, NiNPs caused more severe alveolitis in T-bet^-/-^ mice as compared to wild-type mice. We also observed that MWCNTs, which contain as much as 5% Ni as residual catalyst from the manufacturing process [20], caused significant mucous cell metaplasia and increased CCL2, albeit to a lesser extent than NiNPs. These findings indicate that T-bet is an important regulator of lung injury and remodeling after exposure to either NiNP or MWCNTs, and that the effect of the MWCNTs used in this study may be due to residual Ni catalyst.

T-bet-deficient (T-bet^-/-^) mice have been reported to spontaneously exhibit characteristics resembling the pathophysiology seen in human asthma without the need for allergen sensitization and challenge [9,24]. However, we did not observe obvious spontaneous pathologic changes in airway remodeling (i.e., *mucous cell metaplasia or airway fibrosis) in T-bet^-/-^ mice that were not exposed to NiNPs, but did find spontaneously increased numbers of eosinophils in the BALF from T-bet^-/-^ mice, which is characteristic of allergic asthma. Exposure to NiNPs caused exaggerated asthma-like airway pathology in the T-bet^-/-^, but not WT, suggesting that T-bet^-/-^ mice are ‘primed’ for a Th2-mediated allergic response to NiNPs. The absence of T-bet allows naïve Th cells to differentiate into Th2 cells due to the lack of IFN-γ expression that is produced by CD4^+^ cells when T-bet is present [8]. While Th2 cells are not the only subset of Th cells that mediate allergic lung inflammation, they still contribute to roughly 50% of the mechanisms regulating chronic airway remodeling in the asthmatic population [5,25]. In addition, innate lymphoid cells (ILCs) are a newly described type of cell that share many functional attributes with effector T cell subsets [10]. ILCs are important at mucosal sites where they regulate epithelial cell homeostasis. Therefore, future studies should focus on ILCs in the exacerbation of asthma by nanoparticles. Interestingly, multiple studies have shown that T-bet polymorphisms are associated with clinical asthma phenotypes, the severity of AHR, and altered responses to inhaled corticosteroids [24,26,27]. Given this information, it would be beneficial to utilize T-bet^-/-^ mice to better understand the mechanisms behind the exacerbation of asthma. While a few researchers have taken advantage of this mouse model to study the effects bacteria and viruses have on disease progression [28,29], to our knowledge our study is the first to investigate the effects of engineered nanoparticles on T-bet^-/-^ mice. It would also be important to determine whether nanoparticles further alter the Th2 cell immune cell profile in T-bet^-/-^ mice to a Th17 response, which has been implicated in severe asthma [30].

Mouse models of asthma susceptibility have been developed using 1) gene deletion (e.g., T-bet^-/-^ mice), 2) sensitization by repeated exposure to an allergen, or 3) a combination of genetic deficiency and allergen challenge. As an example of the second category, we previously reported that MWCNTs manufactured with a Ni catalyst exacerbated airway fibrosis in an ovalbumin mouse model of allergic lung inflammation [20]. Inoue and colleagues also showed that MWCNTs enhanced ovalbumin-induced allergic airway inflammation and further demonstrated that MWCNTs significantly increased allergen (OVA)-specific syngeneic T-cell proliferation [31]. As an example of the third category, susceptibility to MWCNT-induced airway inflammation following ovalbumin challenge was enhanced by the loss of cyclooxygenase-2 (COX-2) knock-out mice, indicating that both environmental and genetic factors are important in susceptibility to nanoparticle-induced lung injury [21]. While models of allergen-induced airway inflammation are useful for addressing the issue of susceptibility to engineered nanoparticles, the T-bet^-/-^ mouse does not require allergen sensitization and offers the advantage of a stable genetic shift towards a Th2 immune response. The stable Th2 phenotype of T-bet^-/-^ mice is important since the major pathologic phenotypes exacerbated by NiNPs (mucous cell metaplasia and alveolitis) were observed at 21 days post-exposure and there was little effect seen on inflammation at 1 day post-exposure. On the other hand, allergen-induced mouse models of asthma are relevant to understanding environmental susceptibility to nanoparticle exposure. While both gene deletion and allergen sensitization mouse models have limitations, using a combination of both types of mouse models is a logical approach for elucidating mechanisms of susceptibility to nanoparticle exposure.

Mucus hypersecretion significantly contributes to airflow obstruction during asthma exacerbations that leads to increased rates of morbidity and mortality. It is the major cause behind fatal asthma by occluding roughly 98% of the airways [32,33]. For that reason, it is important to understand how mucous cell metaplasia and mucin regulation is affected by NiNP exposure. Mucin granules, detected in goblet cells by an AB/PAS stain, were slightly increased in the airways of T-bet^-/-^ mice 1 day after initial NiNP exposure (Figure 1). However, examination of epithelial cells by 21 days showed that NiNPs dramatically increased mucous cell metaplasia in T-bet^-/-^ mice but not WT mice. Additionally, we found that levels of mucin mRNAs, MUC5AC and MUC5B, correlated with the amount of mucin protein in T-bet^-/-^ mice that were exposed to NiNPs 21 days post-exposure (Figure 2). This suggested that the presence of T-bet inhibits NiNP-induced mucous cell metaplasia, thereby reducing mucin production that could lead to airway obstruction. Interestingly, ovalbumin (OVA)-sensitized transgenic mice overexpressing T-bet had significantly reduced goblet cell hyperplasia and mucus hypersecretion [34]. Furthermore, another study focused on OVA sensitization in a T cell targeted, tetracycline-inducible T-bet transgenic mouse bred on a T-bet^-/-^ background. That study showed that multiple features of allergic airway inflammation were diminished once T-bet was induced by doxycycline [24]. A third study revealed that OVA sensitized mice treated with an intranasal delivery of T-bet attenuated goblet cell hyperplasia and eosinophilic inflammation [35]. Therefore, our results supports previous studies where T-bet was found to be protective in reducing airway mucus production.

Exaggerated airway inflammation can also contribute to airflow obstruction during asthma exacerbations [3]. Others have shown that micron-sized nickel, NiNPs, and their related compounds have the ability to cause pulmonary inflammation following exposure [19,22,36]. Our data show that acute NiNP-induced acute inflammation at 1 day was localized at alveolar duct bifurcations (ADB) where NiNPs accumulated (Figure 4). However, there was not a quantitative difference in lung inflammation at ADB between NiNP-exposed WT and T-bet^-/-^ mice as determined by pathology scoring of H&E-stained lung sections. Interestingly, we did observe qualitative differences in inflammatory cell profiles from the BALFs between WT and T-bet^-/-^ mice (Figure 3). For example, we found that T-bet^-/-^ mice had spontaneous eosinophil infiltration as well as an increased number of lymphocytes, similar to a previous report [9]. After NiNP exposure, eosinophilic inflammation was further increased at both 1 and 21 days in T-bet^-/-^ mice, while lymphocyte numbers were increased at 21 days after NiNP exposure. In contrast, WT mice displayed an inflammatory response to NiNPs that was comprised primarily of neutrophils and macrophages. Although it was already known that T-bet deficiency causes lymphocytic and eosinophilic inflammation, we have shown that NiNP exposure exaggerates these responses. Moreover, we also observed that neutrophils were prominent in both genotypes in response to NiNPs. While eosinophils are typical in individuals with mild asthma, a mixed neutrophilic and eosinophilic response is indicative of a more severe asthma phenotype that is more often associated with fatal asthma [3].

In addition to the development of chronic inflammation in the lung, inhalation exposure to metallic nickel and nickel-based particles have also been shown to induce pulmonary fibrosis or chronic alveolitis [13,26,37-39]. We have previously reported that MWCNTs manufactured with a NiNP catalyst exacerbated airway fibrosis in an OVA-induced mouse model of allergic lung inflammation [20]. In the present study, we hypothesized that NiNPs or MWCNTs would exacerbate airway fibrosis in the T-bet^-/-^ mouse model of asthma. Unlike the OVA-induced mouse model, MWCNTs did not significantly increase airway fibrosis or lung collagen content in T-bet^-/-^ mice compared to WT mice. In contrast, NiNPs increased airway fibrosis, yet we did not observe a significant difference between WT and T-bet^-/-^ mice (Figure 5). Alternatively, a significant difference in lung parenchymal lesions (i.e., alveolitis) was observed between the two genotypes in response to NiNPs. In the parenchyma of the lungs of T-bet^-/-^ mice, NiNP exposure caused larger and more abundant parenchymal lesions, indicating that the presence of T-bet inhibits the progression of these parenchymal foci. Previous work has shown that T-bet^-/-^ mice are susceptible to bleomycin-induced pulmonary fibrosis compared to WT mice [40]. We did not observe significant increases in lung collagen protein levels using the Sircol Assay. Moreover, the parenchymal lesions were primarily a mix of inflammatory cells and acellular components within alveolar spaces, coupled with alveolar wall thickening, but contained very little trichrome-positive matrix that is indicative of collagen deposition and fibrosis [41]. Therefore, these parenchymal lesions were defined as chronic alveolitis that could represent a precursor to fibrosis. The development of alveolitis after exposure to NiNPs could be significant in reducing lung function. Also, NiNPs were found within the parenchymal lesions. While we did not quantify absolute levels of NiNPs, an important focus of future studies would be to quantify uptake and clearance of NiNPs in the lungs of mice over time and to better understand whether these nanoparticles traffic to other organ systems.

Monocyte chemoattractant protein-1 (MCP-1/CCL2) is an inflammatory chemokine that has been found to regulate the deposition of collagen, recruitment of immune cells to sites of inflammation, and induction of mucin gene expression in epithelial cells [42,43]. Previous work demonstrated that nickel oxide nanoparticles increased CCL2 protein in the BALF of mice [44]. In agreement with these studies, we observed that NiNPs increased CCL2 levels in the lungs of WT mice and we further showed that CCL2 levels were significantly enhanced in the lungs of T-bet^-/-^ mice (Figure 6). During pulmonary fibrosis, CCL2 stimulates fibroblasts to deposit collagen through a TGF-β1-dependent mechanism while during inflammation acts as a chemoattractant for a variety of immune cells including macrophages, mast cells, eosinophils, and T helper 2 cells [45]. Elevated levels of CCL2 have been found in the BALF and bronchial tissue of individuals with allergic asthma [46,47]. Therefore we hypothesized that CCL2 could be responsible for increased mucous cell metaplasia and parenchymal alveolitis seen in T-bet^-/-^ mice exposed to NiNPs. In order to test this hypothesis, T-bet^-/-^ mice were treated with either anti-CCL2 mAb or an IgG2B Isotype Control mAb before and after NiNP exposure. Neutralization of CCL2 in allergen-induced models of asthma has been shown to decrease AHR, inflammation, and macrophage infiltration [48,49]. NiNP-induced lung inflammation was not affected in mice treated with anti-CCL2 mAb (data not shown). Although CCL2 is neutralized, other ligands that bind the CCL2 receptor, CCR2, such as MCP-5 (CCL12) and MCP-3 (CCL7) could function to attract macrophages and eosinophils and thereby regulate inflammation [50,51]. CCR2^-/-^ mice that have decreased amounts of CCL2 from anti-CCL2 gene therapy treatment are protected against bleomycin-induced pulmonary fibrosis [52,53]. While we did not observe significant interstitial fibrosis in T-bet^-/-^ mice treated with NiNPs, we did observe chronic alveolitis and found that anti-CCL2 treatment only caused a slight decrease in alveolitis. The most significant finding was that the anti-CCL2 neutralizing antibody delivered to T-bet^-/-^ mice resulted in enhanced NiNP-induced mucous cell metaplasia (Figure 7). In addition, the anti-CCL2 neutralizing mAb enhanced NiNP-induced MUC5AC and MUC5B mRNA expression in T-bet^-/-^ mice. These findings are in agreement with previous studies which demonstrated that CCR2^-/-^ mice have exacerbated Th2-mediated allergic airway responses to either OVA or *Asperigillus* fungal infection and demonstrate enhanced goblet cell hyperplasia [54,55]. Therefore, CCL2 signaling appears to be an important protective mechanism in suppressing mucous cell metaplasia caused by a variety of inhaled agents including nanoparticles. In general, CCL2 has opposing effects in regulating mucous cell metaplasia and alveolitis or fibrosis in response to a variety of environmental agents including NiNPs. The reasons for these disparate roles of CCL2 in lung disease remain to be elucidated and require further study.

## Conclusion

In summary, our results support the hypothesis that T-bet is an important factor in protecting the lung from exacerbation of allergic airway inflammation and alveolitis caused by lung injury from nickel nanoparticles (NiNPs). These findings suggest that individuals with T-bet deficiency, including individuals with asthma, are at greater risk for the development of NiNP-induced airway mucous cell metaplasia and alveolitis. We also found that CCL2 is enhanced in T-bet^-/-^ mice and blocking CCL2 activity with a neutralizing antibody increased NiNP-induced mucous cell metaplasia in these mice, while marginally reducing NiNP-induced alveolitis. Therefore, CCL2 is a potentially important T-bet-regulated chemokine that appears to play a protective role in selectively suppressing nanoparticle-induced *mucus production in the lungs during allergic inflammation.

## Methods

### Animals

Pathogen-free adult male wild-type (WT) or T-bet^-/-^ C57BL/6 mice were obtained from Taconic Farms, Inc. (Germantown, NY) at 6 to 11 weeks of age or The Jackson Laboratory (Bar Harbor, ME) at 8 weeks. Mice were housed in a temperature and humidity controlled facility and given food and water *ad libitum*. All procedures involving animal use were approved by the Institutional Animal Care and Use Committee (IACUC) at North Carolina State University.

### Nickel Nanoparticles (NiNPs)

Nickel nanoparticles (NiNPs) were purchased from Sun Innovations (Fremont, CA) and have been previously characterized [15,56]. They are characterized as spherical in shape with a ~20 nm diameter, having a specific surface area of 40–60 m^2^/g, a metal purity of 99.9%, and insoluble in water. Size and shape have previously been verified by measuring digitized TEM images with Adobe Photoshop and determining pixel length of >100 NiNPs [15]. Furthermore, the oxidation state of these NiNPs is oxidation state (0) zero [56]. Prior to exposure, NiNPs were suspended in a sterile, 0.1% pluronic F-68 (Sigma) in phosphate buffered saline solution and dispersed for two hours using a bath sonicator at room temperature.

### Multiwalled Carbon Nanotubes (MWCNTs)

MWCNTs (Helix Material Solutions, Inc., Richardson, TX) were synthesized by carbon vapor deposition (CVD) with nickel and lanthanum catalysts. Characterization of the size, purity, surface area and elemental composition provided by the manufacturer was verified independently (Millennium Research Laboratories Inc., Woburn, MA) and has been previously reported by our laboratory [20].

### Experimental design

WT and T-bet^-/-^ mice (*n* = 49, Taconic Farms, Inc.) were exposed to NiNPs or MWCNTs at 4 mg/kg or 0.1% Pluronic (Sigma Aldrich, St. Louis, MO) surfactant solution in phosphate-buffered saline by oropharyngeal aspiration under an isoflurane anesthetic, as previously described [57]. On day 1 or day 21 after initial NiNP exposure, mice were euthanized via intraperitoneal injection of Fatal Plus (Vortech Pharmaceuticals, Dearborn, MI). Lungs were lavaged with Dulbecco’s phosphate-buffered saline (DPBS) and bronchoalveolar lavage fluids (BALF) were collected for ELISA and cell differentials. The middle and caudal lobes of the right lung, as well as the heart, spleen, and a section of the liver, were stored in RNAlater®, according to the manufacturer’s instructions (Ambion, Austin, TX), and used for TaqMan® quantitative real-time RT-PCR analysis. The cranial lobe of the right lung was flash frozen in liquid nitrogen and stored at -80°C for collagen determination and protein evaluation. The left lung was infused with 10% neutral buffered formalin, fixed for 24 hours, transferred to 70% ethanol, and embedded in paraffin. Three cross-sectional sections of tissue were cut and processed for histopathology with a Masson’s trichrome, hematoxylin & eosin (H&E), or Alcian blue/periodic acid-Schiff (AB/PAS) stain.

### Monoclonal antibody administration

A second group of WT and T-bet^-/-^ mice (*n* = 20, The Jackson Laboratory) were treated with either a rat IgG2B Isotype Control antibody (MAB0061, *n* = 7, R&D Systems, Minneapolis, MN) or mouse anti-CCL2 mAb (MAB479, *n* = 8, R&D Systems) by intraperitoneal (i.p.) injection before and after being exposed to 0.1% pluronic solution of NiNPs as described above. Mice received either IgG2B (25 μg) or anti-CCL2 (25 μg) mAb by i.p. injection on days 4, 8, 12, 16, 20, 23, 28, and 32. They were exposed to either the pluronic solution or NiNPs on day 14 and euthanized via intraperitoneal injection of Fatal Plus 21 days after NiNP exposure on day 35. Samples were collected as described in the *Experimental Design*. Mice used in the antibody study were from The Jackson Laboratory (Bar Harbor, ME).

### Bronchoalveolar lavage and cytology

Lungs were serially lavaged three times with 0.5 mL of DPBS and combined. An aliquot from each sample was immediately used to analyze differential cell counts while the remaining sample was stored at -80°C. A Thermo Scientific Cytospin® 4 Cytocentrifuge (Thermo Fisher Scientific, Waltham, MA) was used to plate cells from the BALF of each animal onto glass slides. Samples were then fixed and stained with the Diff-Quik® Stain Set (Dade Behring Inc, Newark, DE). Differential cell counts were performed on at least 500 cells per sample and represented as the mean ± SEM per exposure group.

### Lung fixation and histopathology

At necropsy, the left lungs were pressure-infused intratracheally at 30 cm H_2_O with 10% neutral-buffered formalin. Lungs were fixed for approximately 48 hours and then transferred to 70% ethanol. Three cross-sectional portions of the left lung were embedded in paraffin, sectioned at 5 μm, and stained with hematoxylin and eosin (H&E), Masson’s trichrome, or alcian-blue periodic acid Schiff (AB-PAS) stain by established methods.

### Pathology scoring of lungs

#### Acute inflammation and chronic alveolitis

Three sections of lung, one each from the cranial, middle, and caudal portions of the left lung lobe from each mouse were selected and photographed according to unbiased criteria [58] and evaluated in a blinded fashion as previously described [59]. Those from the 1 day time point were scored for inflammation and those from the 21 day time point for fibrosis or alveolitis. The inflammation scores reflect the averages of the scores for the number of polymorphonuclear cells, the number and size of intra-alveolar macrophage aggregates (these macrophages were typically laden with nanoparticles), and alveolar wall thickening. This average score was then adjusted for the number of nanoparticles present by dividing the average score by the relative score of the number of nanoparticles present to give an adjusted average score. Only the lungs from the 21 day time point were scored for fibrosis or alveolitis. The lungs were scored for the amount of collagen present (based on Masson’s trichrome-stained sections), the thickness of the alveolar walls, and the number of fibroblast-like cells associated with the particle-associated lesions. These scores were averaged and the average scores were then divided by the relative scores for the amount of nanoparticles present in the lungs to give an adjusted average score for each animal. All scores were relative scores on a scale from 1–5, with 1 representing the levels of these parameters in the PBS control group, 2 representing minimal change, 3 representing mild change, 4 representing moderate change, and 5 representing severe change. In these numeric categories, the term ‘change’ refers to the presence of intra-alveolar inflammatory cells and extracellular matrix, and alveolar wall thickening. The scores from all three observers were averaged and the data was presented as the mean value ± SEM of the inflammation score of 5–7 animals for each dose group. The scoring system for acute inflammation at 1 day and parenchymal fibrosis/alveolitis at 21 days used in this study is an unbiased method that we have employed previously [59] and is derived from a simple unbiased method of estimating pulmonary fibrosis on a numerical scale [60].

#### Mucous cell metaplasia

Airway mucin production was analyzed using a previously established protocol [61]. Photomicrographs of AB/PAS stained lung sections were measured and quantified using ImageJ version 1.44o (National Institutes of Health). All bronchioles measured for mucous cell metaplasia displayed cross-sectional oval or elliptical profiles with a diameter between 100–200 micrometers [20]. Cells that were positively stained for AB/PAS were identified by the de-convolution module using a threshold method and then calibrated for measurement in microns. Data was expressed as the percentage of epithelial area positively stained for AB/PAS divided by the total epithelial area (%PAS+/Total Area).

#### Airway collagen deposition

Thickness of collagen surrounding airway bronchioles was quantified using Adobe Photoshop CS5 (Adobe Systems, Inc., San Jose, CA) according to a previously published procedure [62]. Photomicrographs of trichrome-stained sections were captured using the 10× objective on an Olympus BX41 microscope (Olympus America, Inc., Canter Valley, PA) and digitized. All bronchioles measured for airway fibrosis displayed cross-sectional oval or elliptical profiles with a diameter between 100–200 micrometers [20]. Airway collagen area was measured and corrected for length of basement membrane (area/perimeter ratio) using the lasso tool in Adobe Photoshop. At least three airways per animal were analyzed in a blinded, random manner and expressed as the mean ± SEM of 5–7 animals per treatment group per time point.

### ELISA

Quantikine ELISA kits (R&D Systems) were used to assay protein levels of IL-13 or CCL2 in BALF. Samples were assayed according to kit instructions and absorbance values were measured by the Multiskan FC microplate spectrophotometer microplate reader (Thermo Fisher Scientific).

### Taqman quantitative real-time RT-PCR

One-step, TaqMan® quantitative real time RT-PCR (qRT-PCR) was performed to quantify gene expression of our target genes in lung tissue 1 and 21 days after NiNP exposure. Total RNA was extracted and purified from the right middle and caudal lobes of each lung using an RNeasy™ Fibrous Tissue Mini Kit (Qiagen, Valencia, CA). RNA concentrations were determined by the Nanodrop® 1000 spectrophotometer and samples were normalized to a final concentration of 25 ng/μl. qRT-PCR was performed using reagents from the SuperScript® III Platinum® One-Step qRT-PCR Kit (Invitrogen, Grand Island, NY) on the StepOne® Plus instrument (Applied Biosystems, Foster City, CA). A comparative C_T_ method was used to quantify target gene expression for Mucin 5 AC (MUC5AC, Mm01276718_m1), Mucin 5B (MUC5B, Mm00466391_m1), CCL2/MCP-1 (Mm00441242_m1), IL-13 (Mm00434204_m1), and col1a2 (Mm00483888_m1), normalized against the endogenous control β-2 Microglobulin (B2M, Mm00437762_m1) and measured relative to the vehicle-treated control groups. Each individual sample was analyzed in duplicate while the StepOne® Plus software calculated relative quantitation values that were expressed as fold-change over controls.

### Sircol assay for soluble collagen

Tissue from the right cranial lobe of each mouse lung were weighed between 10–50 mg, suspended in 1 mL of DPBS, and homogenized for 60 seconds with a Tissuemiser® homogenizer (Thermo Fisher Scientific). Soluble collagen was then measured following the Sircol® Soluble Collagen Assay kit (Biocolor Ltd., Carrickfergus, UK) protocol as previously described [20]. Additionally, total protein was analyzed, prior to collagen extraction, using a BCA Protein Assay kit (Thermo Fisher Scientific). Data were expressed as μg of soluble collagen per mg of total protein.

### Statistics

All graphs were constructed and statistical analysis was performed using GraphPad Prism software version 5.00 (GraphPad Software, Inc., San Diego, CA). Data are the expressed as mean values ± SEM of 5–6 (Control) or 6–7 (NiNP) animals per genotype. Samples were assayed in duplicate for quantitative real-time RT-PCR, triplicate for ELISA, or as mentioned. Groups were compared with one-way ANOVA with a *post hoc* Tukey, unpaired, two-tailed Student’s t-test, or two-way ANOVA with a Bonferroni test. A value of *p* ≤ 0.05 was considered significant.

## Abbreviations

ENM: Engineered nanomaterial; NiNP: Nickel nanoparticle; Th cell: T helper cell; T-bet: T-box transcription factor TBX21; BALF: Bronchoalveolar lavage fluid; ROS: Reactive oxygen species; HIF: Hypoxia-inducible factor; Ni: Nickel; WT: Wild-type; AB/PAS: Alcian blue/periodic acid-Schiff.

## Competing interests

The authors declare that they have no competing interests.

## Authors’ contributions

EEG and JCB planned and developed the experimental design. EEG, AJT, BCS, EAT and JCB performed experimental procedures and collected data. EEG analyzed the data, wrote the manuscript, and prepared all figures. JCB edited text and figures. All authors read and approved the final manuscript.

## Supplementary Material

Additional file 1 **Comparison of MWCNTs and NiNPs in the induction of airway mucous cell metaplasia and MUC5AC mRNA levels in the lungs of WT and T-bet**^**-/- **^**mice.** A) Quantification of mucus producing cells at 21 days post-exposure determined using ImageJ analysis software (NIH). Data presented as the percentage of AB/PAS-positive stained area per total area. Asterisks directly above bars (**p < 0.01 or ***p < 0.001) indicate comparison to the control group of the same genotype. Also, a significant difference (**p < 0.01) between MWCNT and NiNP in T-bet^-/-^ mice is indicated. All data represent mean values ± SEM of at least three measurements per lung of 5–7 mice per exposure group. B) Taqman quantitative real-time RT-PCR was used to measure changes in whole lung mRNA levels of MUC5AC Values are means ± SEM (*n* = 5–7 animals/group). Asterisks directly above bars (**p < 0.01 or ***p < 0.001) indicate comparison to the control group of the same genotype. Also, a significant difference (**p < 0.01) between MWCNT and NiNP in T-bet^-/-^ mice is indicated. MWCNT induction of MUC5AC was not significant ‘ns’ (p > 0.05). Click here for file

Additional file 2 **Comparison of MWCNTs and NiNPs for causing eosinophilia or neutrophilia in the lungs of WT and T-bet**^**-/- **^**mice.** MWCNT or NiNP were delivered to the lungs of mice by OPA followed by collection of lung BALF 1 day post-exposure. Relative numbers of A) eosinophils and B) neutrophils were quantified by differential cell counting. Data are the mean values ± SEM out of a total of 500 cells counted per animal for 5–7 animals per dose group at 20X magnification. Asterisks above bars (**p < 0.01 or ***p < 0.001) indicate comparison to the saline control group of the same genotype. Significant differences (*p < 0.05, **p < 0.01, ***p < 0.001) between MWCNT and NiNP in WT or T-bet^-/-^ mice are indicated. The effect of MWCNTs on eosinophilia in T-bet^-/-^ mice was not significant ‘ns’ (p > 0.05) compared to saline control. Click here for file

Additional file 3 **Comparison of NiNPs and MWCNTs in the induction of CCL2 protein levels in BALF from the lungs of WT and T-bet**^**-/- **^**mice.** CCL2 protein in BALF was analyzed by ELISA after 1 day. Asterisks above bars (*p < 0.05 or ***p < 0.001) indicate comparison to the saline control group of the same genotype. Significant differences (**p < 0.01, ***p < 0.001) between MWCNT and NiNP in WT or T-bet^-/-^ mice are indicated. Data are the mean values ± SEM (*n* = 5–7 animals/group). Click here for file

Additional file 4 **Total lung soluble collagen expression in the lungs of WT and T-bet**^**-/- **^**mice in response to NiNP exposure.** A) Col1a2 mRNA expression levels were measured by qRT-PCR in whole lung tissue at 1 and 21 days after initial exposure (nd, not detectable). B) Soluble collagen content, μg/mg of protein, was measured from whole lung homogenates using the Sircol Assay kit. Data are mean values ± SEM (*n* = 5–7 animals/group). *p < 0.05, **p < 0.01, ***p < 0.001 as compared to the control group of the same genotype or as indicated. Click here for file

Additional file 5 **IL-13 mRNA and protein expression 1 and 21 day post exposure in the lungs of WT and T-bet**^**-/- **^**mice.** A) Levels of IL-13 mRNA was measured in whole lung tissue by qRT-PCR at 1 and 21 days after exposure. B) Protein expression of IL-13 in the BALF was analyzed by ELISA at 1 or 21 days post exposure. All data represent mean values ± SEM. *p < 0.05, **p < 0.01, ***p < 0.001 as compared to the control group of the same genotype or as indicated (*n* = 5–7 animals/group). Click here for file
