# Supplementary figures and images for: Nickel Nanoparticles cause exaggerated lung and airway remodeling in mice lacking the T-box transcription factor, TBX21 (T-bet)
Source: Part Fibre Toxicol. 2014 Feb 6;11:7. doi: 10.1186/1743-8977-11-7 (PMC3931667; doi:10.1186/1743-8977-11-7)

# Additional File 1

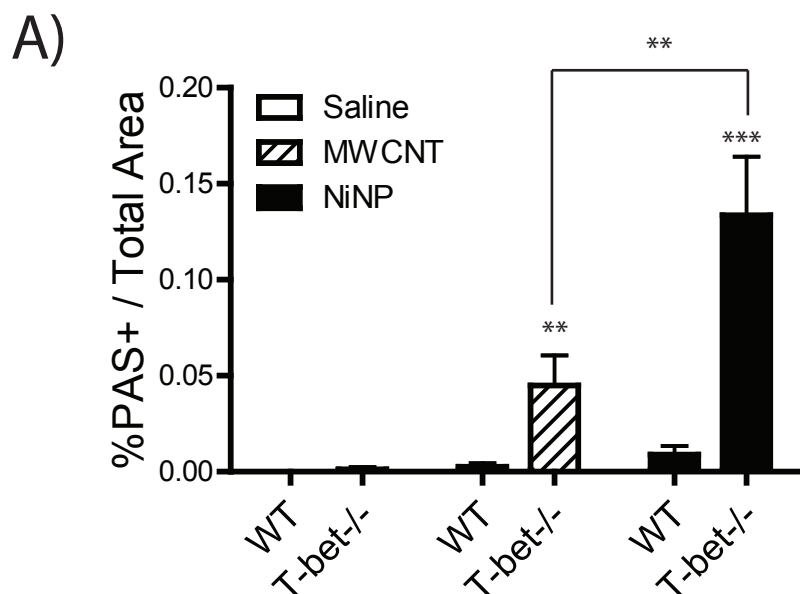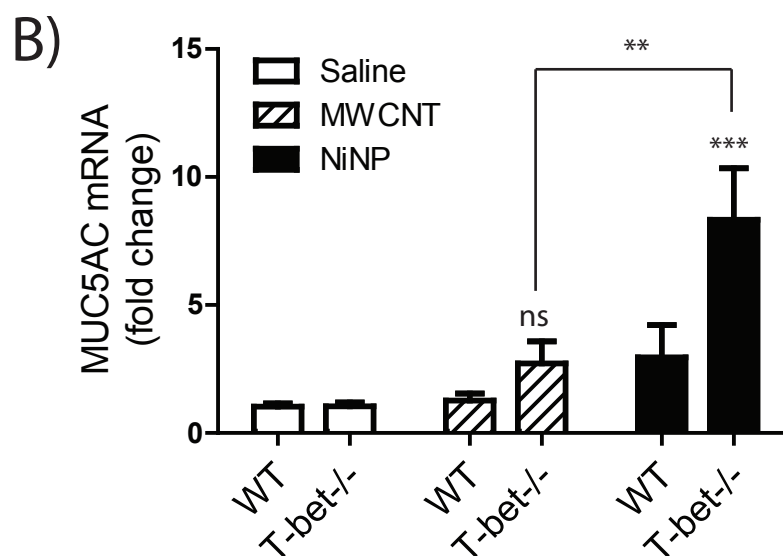

Supplement: Additional file 1 — Comparison of MWCNTs and NiNPs in the induction of airway mucous cell metaplasia and MUC5AC mRNA levels in the lungs of WT and T-bet -/- mice. A) Quantification of mucus producing cells at 21 days post-exposure determined using ImageJ analysis software (NIH). Data presented as the percentage of AB/PAS-positive stained area per total area. Asterisks directly above bars (**p < 0.01 or ***p < 0.001) indicate comparison to the control group of the same genotype. Also, a significant difference (**p < 0.01) between MWCNT and NiNP in T-bet-/- mice is indicated. All data represent mean values ± SEM of at least three measurements per lung of 5–7 mice per exposure group. B) Taqman quantitative real-time RT-PCR was used to measure changes in whole lung mRNA levels of MUC5AC Values are means ± SEM (n = 5–7 animals/group). Asterisks directly above bars (**p < 0.01 or ***p < 0.001) indicate comparison to the control group of the same genotype. Also, a significant difference (**p < 0.01) between MWCNT and NiNP in T-bet-/- mice is indicated. MWCNT induction of MUC5AC was not significant ‘ns’ (p > 0.05). [file 1743-8977-11-7-S1.pdf]

## Additional File 2

A)

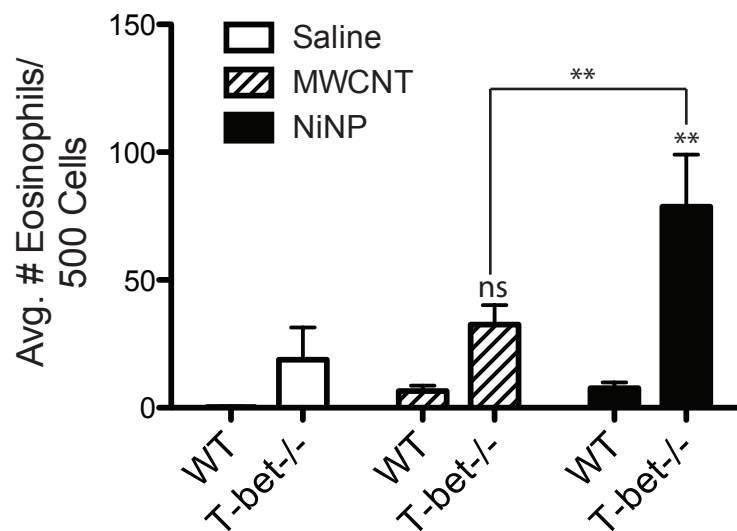

B)

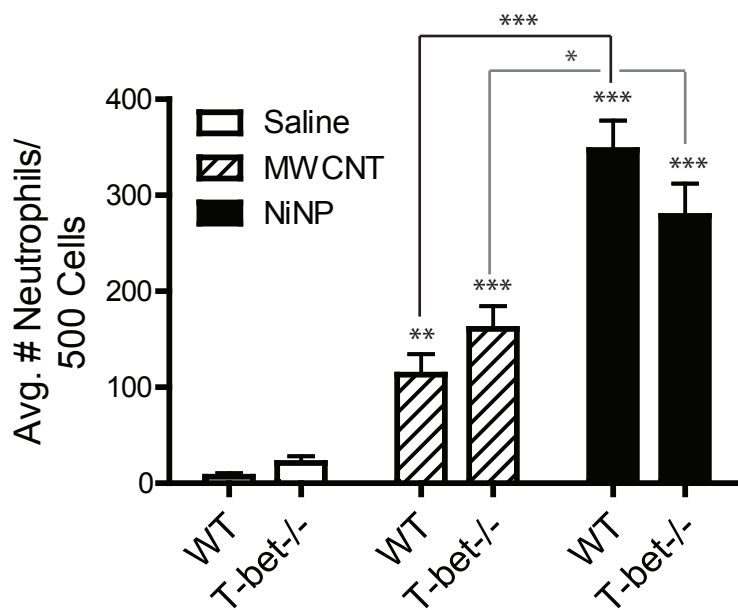

Supplement: Additional file 2 — Comparison of MWCNTs and NiNPs for causing eosinophilia or neutrophilia in the lungs of WT and T-bet -/- mice. MWCNT or NiNP were delivered to the lungs of mice by OPA followed by collection of lung BALF 1 day post-exposure. Relative numbers of A) eosinophils and B) neutrophils were quantified by differential cell counting. Data are the mean values ± SEM out of a total of 500 cells counted per animal for 5–7 animals per dose group at 20X magnification. Asterisks above bars (**p < 0.01 or ***p < 0.001) indicate comparison to the saline control group of the same genotype. Significant differences (*p < 0.05, **p < 0.01, ***p < 0.001) between MWCNT and NiNP in WT or T-bet-/- mice are indicated. The effect of MWCNTs on eosinophilia in T-bet-/- mice was not significant ‘ns’ (p > 0.05) compared to saline control. [file 1743-8977-11-7-S2.pdf]

# Additional File 3

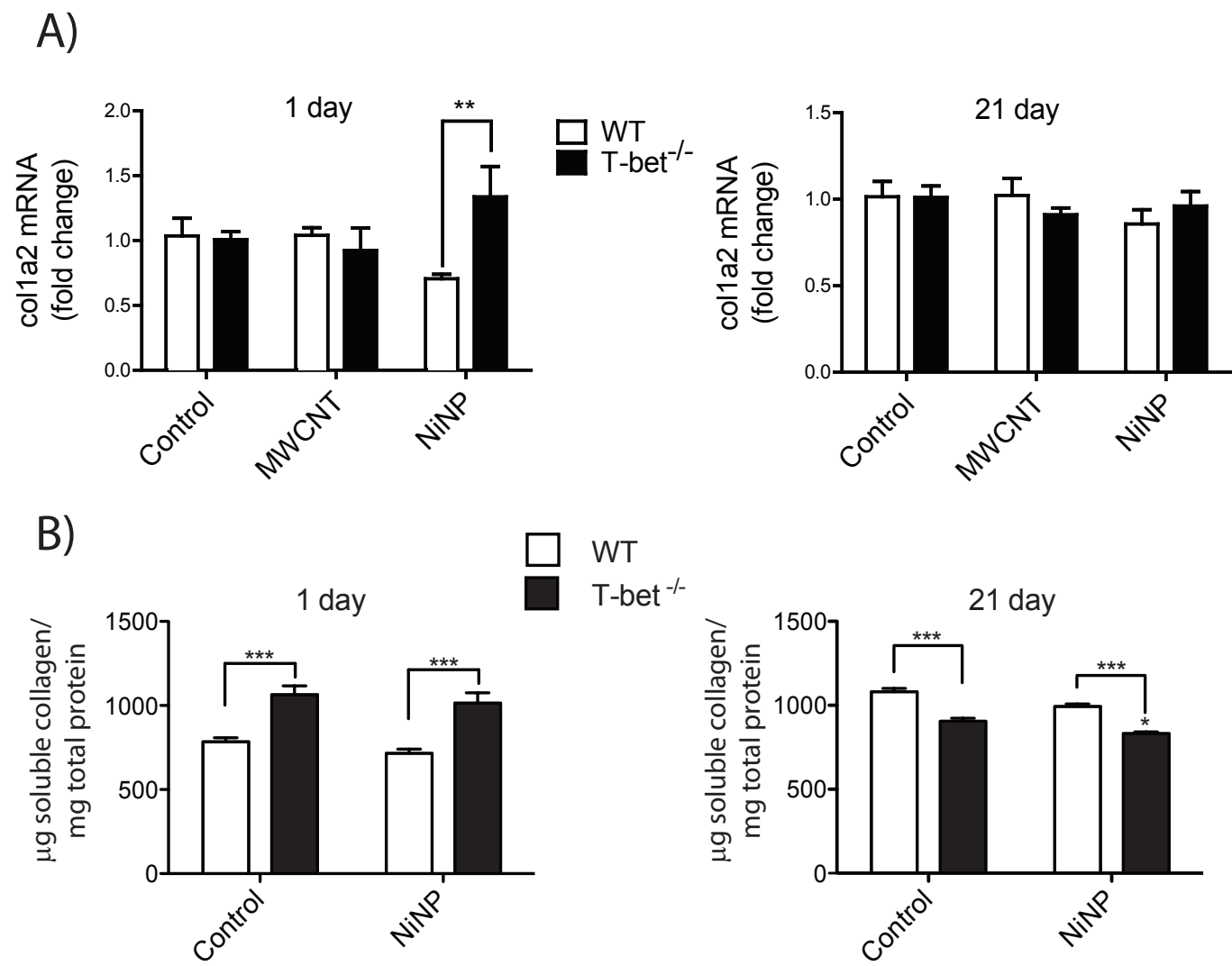

Supplement: Additional file 3 — Comparison of NiNPs and MWCNTs in the induction of CCL2 protein levels in BALF from the lungs of WT and T-bet -/- mice. CCL2 protein in BALF was analyzed by ELISA after 1 day. Asterisks above bars (*p < 0.05 or ***p < 0.001) indicate comparison to the saline control group of the same genotype. Significant differences (**p < 0.01, ***p < 0.001) between MWCNT and NiNP in WT or T-bet-/- mice are indicated. Data are the mean values ± SEM (n = 5–7 animals/group). [file 1743-8977-11-7-S3.pdf]

Additional File 4

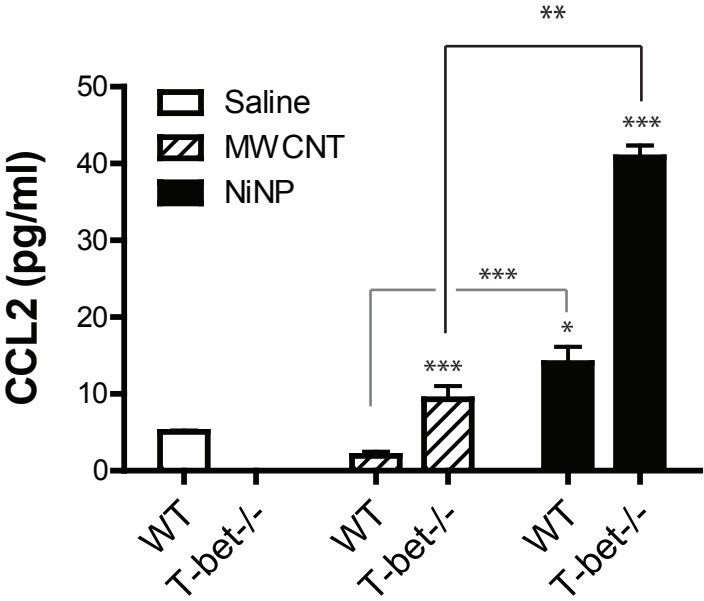

Supplement: Additional file 4 — Total lung soluble collagen expression in the lungs of WT and T-bet -/- mice in response to NiNP exposure. A) Col1a2 mRNA expression levels were measured by qRT-PCR in whole lung tissue at 1 and 21 days after initial exposure (nd, not detectable). B) Soluble collagen content, μg/mg of protein, was measured from whole lung homogenates using the Sircol Assay kit. Data are mean values ± SEM (n = 5–7 animals/group). *p < 0.05, **p < 0.01, ***p < 0.001 as compared to the control group of the same genotype or as indicated. [file 1743-8977-11-7-S4.pdf]

# Additional File 5

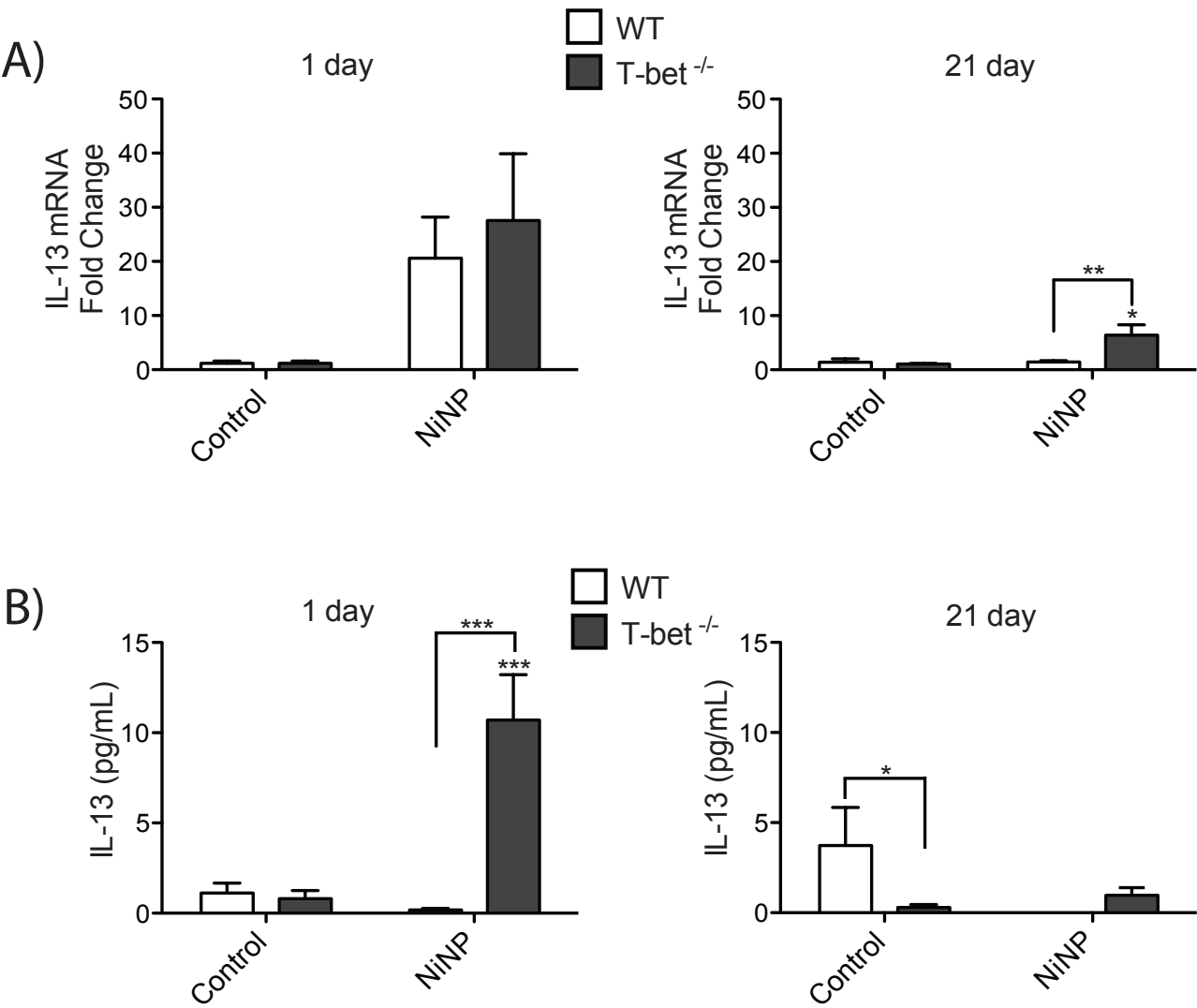

Supplement: Additional file 5 — IL-13 mRNA and protein expression 1 and 21 day post exposure in the lungs of WT and T-bet -/- mice. A) Levels of IL-13 mRNA was measured in whole lung tissue by qRT-PCR at 1 and 21 days after exposure. B) Protein expression of IL-13 in the BALF was analyzed by ELISA at 1 or 21 days post exposure. All data represent mean values ± SEM. *p < 0.05, **p < 0.01, ***p < 0.001 as compared to the control group of the same genotype or as indicated (n = 5–7 animals/group). [file 1743-8977-11-7-S5.pdf]
